# Comorbidity in elderly cancer patients in relation to overall and cancer-specific mortality

**DOI:** 10.1038/bjc.2012.46

**Published:** 2012-02-21

**Authors:** T L Jørgensen, J Hallas, S Friis, J Herrstedt

**Affiliations:** 1 Department of Oncology, Odense University Hospital, Strandboulevarden 29, 5000 Odense C, Denmark; 2 Institute of Public Health, IPH, Research Unit of Clinical Pharmacology, University of Southern Denmark, Winsløwparken 19^2^, 5000 Odense C, Denmark; 3 Institute of Cancer Epidemiology, Danish Cancer Society, Strandboulevarden 49, 2100 Copenhagen Ø, Denmark

**Keywords:** aged, elderly, comorbidity, survival

## Abstract

**Background:**

Aims of this study were to describe the prevalence of comorbidity in newly diagnosed elderly cancer cases compared with the background population and to describe its influence on overall and cancer mortality.

**Methods:**

Population-based study of all 70+ year-olds in a Danish province diagnosed with breast, lung, colorectal, prostate, or ovarian cancer from 1 January 1996 to 31 December 2006. Comorbidity was measured according to Charlson's comorbidity index (CCI). Prevalence of comorbidity in newly diagnosed cancer patients was compared with a control group by conditional logistic regression, and influence of comorbidity on mortality was analysed by Cox proportional hazards method.

**Results:**

A total of 6325 incident cancer cases were identified. Elderly lung and colorectal cancer patients had significantly more comorbidity than the background population. Severe comorbidity was associated with higher overall mortality in the lung, colorectal, and prostate cancer patients, hazard ratios 1.51 (95% CI 1.24–1.83), 1.41 (95% CI 1.14–1.73), and 2.14 (95% CI 1.65–2.77), respectively. Comorbidity did not affect cancer-specific mortality in general.

**Conclusion:**

Colorectal and lung cancer was associated with increased comorbidity burden in the elderly compared with the background population. Comorbidity was associated with increased overall mortality in elderly cancer patients but not consistently with cancer-specific mortality.

Because of the aging population, the number of elderly cancer patients (⩾70 years) has increased rapidly during the last two decades (Engholm *et al*, 2011). Several reports have shown a high prevalence of comorbid conditions among elderly cancer patients (Ogle *et al*, 2000; Koroukian *et al*, 2006; Wedding *et al*, 2007), but few studies have compared the prevalence of comorbidity in people with and without cancer. Furthermore, results have been conflicting; some studies have reported a clear association between a diagnosis of cancer and increased comorbidity (Smith *et al*, 2008), whereas others have not (Zeber *et al*, 2008; Driver *et al*, 2010).

A high level of comorbidity is associated with lower survival of cancer patients (Yancik *et al*, 1998; Lund *et al*, 2008; Tetsche *et al*, 2008; Patniak *et al*, 2011). Obviously, this could be explained by excess mortality related to the comorbid conditions. There are a number of reasons why cancer-specific mortality may be elevated as a consequence of non-malignant comorbidity: for example, due to suboptimal antineoplastic treatment of these patients (Hurria *et al*, 2003; Janssen-Heijnen *et al*, 2005; Koppie *et al*, 2008) and/or to an increased treatment toxicity resulting in reduced treatment adherence. Indeed, one study found all levels of comorbidity in lung cancer patients to be associated with increased toxicity and reduced total dose of chemotherapy, and comorbidity was found to be predictive of a decrease in overall survival whereas age itself was not (Asmis *et al*, 2008). Treatment toxicity could also influence the prognosis. Furthermore, comorbidity has been associated with delay of cancer diagnosis and hence more advanced disease stage at diagnosis (Bjerager *et al*, 2006). Few studies have focused on cancer-specific mortality and its relation to comorbidity.

The aims of this study were to describe the prevalence of comorbidity in newly diagnosed elderly cancer cases compared with the background population and to describe the influence of comorbidity on overall and cancer-specific mortality.

## Materials and methods

For the description of comorbidity of cancer cases, we conducted a population-based case–control study of all inhabitants, aged 70 years and more, of Funen County (population 480 000), Denmark, who were diagnosed with breast, lung, colorectal, prostate, or ovarian cancer from 1 January 1996 to 31 December 2006. The population of Funen constitutes ∼9% of the Danish population, and validation studies have previously shown that Funen is representative of the entire population of Denmark (Gaist *et al*, 1997). The second part of the study, describing overall and cancer-specific mortality according to level of comorbidity, was designed as a retrospective cohort study using the cancer subjects only.

### Data sources

We obtained data from four registers: The Danish Cancer Register (DCR), the Funen County Patient Administrative System (FPAS), Odense Pharmacoepidemiologic Database (OPED), and the Danish Causes of Death Register (DCDR). Linkage between these registers was carried out using the personal identification number (PIN); a unique identifier of all Danish citizens assigned by the Central Population Register since 1968. The PIN includes date of birth and gender, and allows an accurate linkage between population-based registers (Pedersen *et al*, 2006).

The DCR has recorded incident cases of cancer in Denmark since 1943 and has been shown to have accurate and almost complete capture of cancer cases (Storm *et al*, 1997). The cancer diagnoses in the DCR are coded by the 10th revision of the International Classification of Diseases (ICD-10). Among other variables, it includes the PIN, date of cancer diagnosis, method of verification, diagnosis, histological classification, and date of death.

The FPAS has recorded all discharge diagnoses of non-psychiatric hospital admissions in Funen since 1973. From 1995, all outpatient contacts have been recorded as well. Diagnoses in the FPAS are coded by the ICD-8 until December 1993, hereafter by the ICD-10. Secondary care, inpatient or outpatient, is provided almost exclusively by the national health services, and, thus, this register covers virtually all hospital contacts in Funen County.

The prescription database OPED has collected data on all reimbursed prescriptions in Funen since 1990. The national health services partly reimburse most prescription drugs for all Danish inhabitants independently of private insurances. Drugs are classified according to the Anatomic Therapeutic Chemical (ATC) classification system, developed by the WHO (WHO, 2003). Each record in OPED contains the PIN of the patient, the date of purchase, the pharmacy, the prescriber, and a full account of what has been dispensed, including the brand name, the ATC code, dose unit, and quantity. The prescribed daily dose and the indication for prescribing are not recorded in the database. Odense Pharmacoepidemiologic Database also contains a demographic module which holds information on residency, migrations, and death of all citizens of Funen (Hallas and Nissen, 1994; Hallas, 2001).

Since 1943, the DCDR has recorded data on death certificates of all the Danish citizens. The register holds information on the PIN, date of death, main cause of death, and on up to four contributory causes of death, coded according to the ICD-10 since 1994 (Juel and Helweg-Larsen, 1999).

### Cancer cases and controls

We identified 6325 cases with a first time diagnosis of breast, lung, colorectal, prostate, or ovarian cancer during the period 1 January 1996 to 31 December 2006. Breast, lung, colorectal, and prostate cancer were selected since these cancer types constitute the four most frequent cancer types in Denmark. Ovary cancer was included since a future nationwide study of this cancer was planned simultaneously with the present study. Cases were assigned with an index date, which was the date of cancer diagnosis.

Controls were extracted from OPED's demographic module by use of a risk set sampling technique (Langholz and Goldstein, 1996). For each case, we randomly selected four controls among all residents of Funen who matched the case by birth year and gender, and who did not have a diagnosis of cancer at the time the corresponding case was diagnosed (index date). One 103-year-old colorectal cancer case only had three eligible controls and, thus, our final control/case ratio deviated slightly from 4 : 1. Controls were eligible as cases at a later point in the study period.

### Comorbidity

Comorbidity was described according to Charlson's comorbidity index (CCI) (Charlson *et al*, 1987). The CCI is a weighted index that takes into account both the number and the seriousness of comorbid diseases, and it was originally validated in the breast cancer patients. The index is based on 19 chronic conditions, each with an assigned weight from 1–6 according to the relative risk of dying within 1 year. Four of the 19 conditions described in the CCI are related to malignant disease and these were excluded from our analyses to allow a balanced comparison between subjects with and without cancer diagnoses. The CCI has previously been adapted for use with ICD-10 administrative data (Nuttall *et al*, 2006), and the index has been validated specifically in elderly cancer patients (Extermann, 2000). We divided the CCI score (CCIS) into three groups: CCIS 0=no comorbidity, CCIS 1–2=low to moderate comorbidity, and CCIS ⩾3=severe comorbidity.

As some patients were diagnosed and treated for type 2 diabetes (diabetes mellitus, DM) solely in primary care, we classified all users of antidiabetics (ATC codes A10A and A10B) as having a diagnosis of DM, which yielded additional 367 patients. Likewise, users of drugs for obstructive airway diseases, ATC code R03 (but not beta-adrenergics alone), were encoded as having chronic pulmonary disease (CPD) even if they did not have a CPD diagnosis in the FPAS (yielding additional 986 patients). Finally, we encoded all subjects as having dementia if they had the diagnosis in FPAS or if they were using anti-dementia drugs, ATC code N06D (24 additional patients). As we have earlier found that drug use among elderly cancer patients increases significantly during the past 6 months before diagnosis (Jorgensen *et al*, 2012), we used the period of 6–10 months before index date to assess drug use, defined as using one or more prescription medication during this period.

### Death and censoring

We used the primary cause of death from the DCDR as outcome of death. The diagnoses were divided into two subgroups; death from the primary cancer and death from other causes. A total of 1416 cases had a malignant cause of death diagnosis other than the original cancer diagnosis. These cases were controlled manually (TLJ), and 26 were found to have died from a secondary malignant disease. In 96 observations, the dates differed between the DCR and the DCDR, and in 79 cases, the date of death was only recorded in one of the registers. We believe it is unlikely that study subjects would be registered with a date of death in either register if they had not indeed died and these subjects were treated in the analyses as such. However, for 42 cases with a date of death only from the DCR, we had no information on the cause of death. Twenty-nine of these cases were not censored before death because of the end of follow-up or emigration and were therefore excluded from the mortality analyses Further, we also excluded 38 cases aged 70 years or more who were registered with a cancer diagnosis at the date of death only. After these exclusions, 6287 study subjects were available for analyses of overall mortality, and 6258 for cancer-specific mortality.

### Analysis

We used conditional logistic regression to compute odds ratios (ORs) for the association between low–moderate and severe comorbidity and the five cancer groups. The reference for all of these analyses was no comorbidity.

We analysed the trend in comorbidity for the study period by a logistic regression model with calendar year and case status as explanatory variables. The model was validated by a goodness-of-fit test, comparing the observed proportions with the corresponding predictions from the model. The difference was significant, and an interaction variable between cases status and calendar year was included in the model.

Kaplan–Meier survival analysis by CCIS 0, 1–2, and 3+ was calculated and plotted for each cancer site. Follow-up started on the date of diagnosis and continued until death, emigration, or 31 December 2008, whichever occurred first. In the analyses of cancer-specific mortality, deaths from other causes than the primary cancer were treated as censoring events. To estimate the association between comorbidity and overall and cancer-specific mortality, we used Cox proportional hazards analysis, and the hazard ratios (HRs) calculated were adjusted for age, gender (where relevant), and year of diagnosis. We speculated that the effect of age might be quadratic, not linear, and constructed a variable that equalled the squared value of age. This variable was only significant in analyses of prostate cancer cases, and is included only for this site. The proportional hazards assumption was checked graphically using the Nelson–Aalen estimator and analytically using Schoenfeld residuals.

All statistical analyses were performed using Stata version 11 (StataCorp. LP, College Station, TX, USA).

The study was approved by the Danish Data Protection Agency.

## Results

In the study period, 6325 cases of breast, lung, colorectal, prostate, and ovarian cancer were identified. Median age was 78 years, range 70–103. The three most common comorbidities among cases and controls were CPD, DM, and congestive heart failure. Further characteristics of cases and the 25 299 controls are displayed in Table 1.

**Figure 1:**
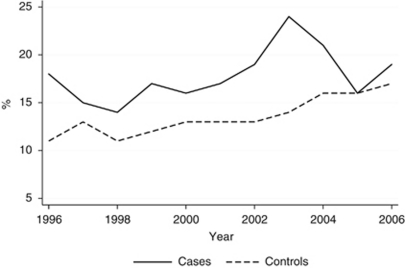
Proportion of cases and controls with CCIS ⩾1 according to study year.

Overall, comorbidity at index date increased modestly during the study period 1996–2006. Overall, OR for having at least one comorbidity was 1.05 (95% CI 1.03–1.07) per year. The increase was similar among elderly cases and controls, OR 1.13 (95% CI 0.90–1.43). The prevalence of comorbidity in the two groups according to study year is shown in Figure 1.

**Figure 2:**
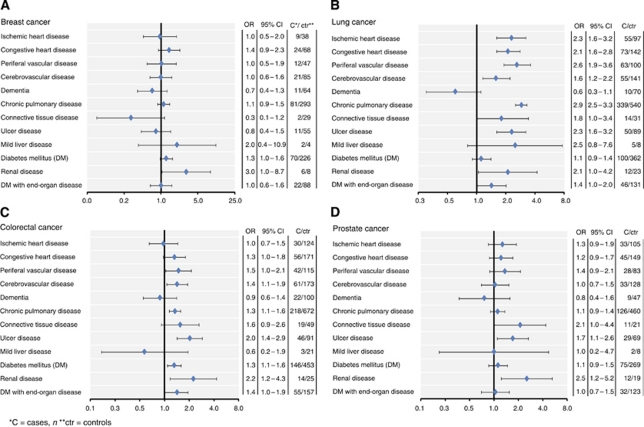
Forest plot of ORs associating Charlson comorbidity items with a diagnosis of cancer according to cancer site.

ORs of all comorbidities according to cancer site are shown in Figures (2A–D). Regardless of cancer site, moderate–severe renal disease was more prevalent in cases than in controls, ORs were 3.00 (95% CI 1.04–8.65), 2.09 (95% CI 1.04–4.19), 2.24 (95% CI 1.16–4.31), and 2.53 (95% CI 1.23–5.20) for breast, lung, colorectal, and prostate cancer, respectively. There were too few diagnoses of moderate to severe renal disease among ovarian cancer cases and controls to provide a valid estimate (data not shown).

Prevalence of morbidity and ORs for the association between CCIS and cancer status according to cancer site are shown in Table 2. Lung cancer cases had more comorbidity than controls in all age groups. Cases had more of all comorbidities, except diabetes and dementia, which appeared less frequently than in controls, although the results were not statistically significant (Figure 2B). Cases with colorectal cancer also had more comorbidity than controls, especially ulcer disease, vascular disease, and DM (Figure 2C). Prostate cancer cases especially had more ulcer and connective tissue disease (Figure 2D).

**Figure 3:**
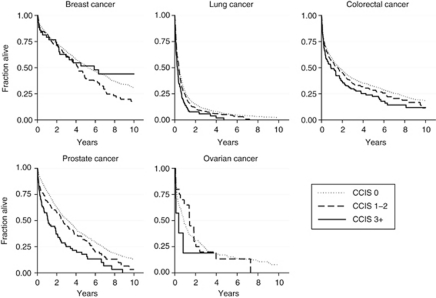
Overall survival according to CCIS and cancer site.

**Figure 4:**
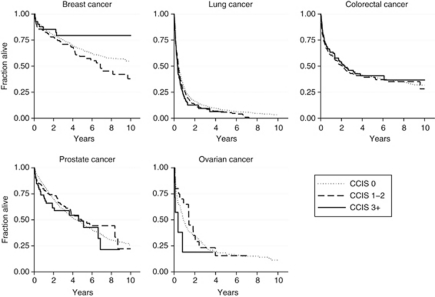
Cancer-specific survival according to CCIS and cancer site.

Cumulative incidence proportions of deaths at 3 months, 1 year, and 5 years, and HRs are shown according to cancer site and comorbidity status for overall and cancer-specific mortality in Tables 3 and 4. Corresponding Kaplan–Meier survival analyses are shown if Figures 3 and 4. Breast cancer cases with CCIS 1–2 had higher overall and cancer-specific mortality than cases with CCIS 0 and CCIS 3+. For lung, colorectal and prostate cancer cases, severe comorbidity was associated with increased overall mortality: HR for CCIS 3+ was 1.51 (95% CI 1.24–1.83), 1.41 (95% CI 1.14–1.73), and 2.14 (95% CI 1.65–2.77), respectively. For cancer-specific mortality, this association was only seen in lung cases, HR 1.29 (95% CI 1.03–1.60). For ovarian cancer cases CCIS was not associated with increased mortality in general.

## Discussion

This population-based study demonstrated an association with frequencies of low–moderate and severe comorbidity and diagnoses of lung and colorectal cancer among elderly individuals. Moderate to severe renal disease was associated with diagnoses of several cancer types. Comorbidity was associated with increased overall mortality across most cancer sites. For cancer-specific mortality, however, the association was only clearly apparent among lung cancer patients.

End-stage renal disease has previously been associated with an increased occurrence of several cancer diseases (Sutherland *et al*, 1977; Inamoto *et al*, 1991; Maisonneuve *et al*, 1999). The reason for this association is not clear, but chronic infections, a compromised immune system, and altered DNA repair have been suggested as possible risk factors (Vamvakas *et al*, 1998).

Breast cancer cases suffered marginally more from DM than controls, and as DM is a known risk factor for the development of breast cancer (Larsson *et al*, 2007), this association may have a causal component. Among breast cancer cases, we found the highest overall and cancer-specific mortality among those with CCIS 1–2, which is in contrast with other studies (Land *et al*, 2011). Sample size was low, however, with only 43 cases having CCIS 3+, so this result might be a chance finding.

Lung cancer cases had significantly more comorbidity than controls. Lung cancer is mainly caused by cigarette smoking and lung cancer patients have a high comorbidity burden caused by other smoking-related diseases such as CPD and cardio-vascular disease (Janssen-Heijnen *et al*, 2005). Smoking was thus an important cause of the comorbidity–lung cancer association. Comorbidity influenced both overall and cancer-specific mortality. Two explanations of this pattern may be offered. First, comorbidity has been found to cause delay of diagnosis of lung cancer (Bjerager *et al*, 2006) and advanced stage of lung cancer at diagnosis is strongly related to increased mortality. Second, the high prevalence of comorbidity in these patients might result in ineligibility for surgery, radiotherapy, and chemotherapy. Indeed, lung cancer patients with two or more comorbid conditions have been reported to be less likely to receive active treatment for their disease (Blanco *et al*, 2008).

The prevalences of vascular disease, CPD, ulcer disease, and DM were higher in colorectal cancer cases than in controls. Life-style factors such as poor diet, obesity, and low physical activity level are risk factors for colorectal cancer as well as of conditions like DM and vascular disease (Libutti *et al*, 2011). Other studies have reported similar results as ours and also found a high prevalence of ulcer and CPD (Yancik *et al*, 1996; Gross *et al*, 2006; Smith *et al*, 2008), whereas some studies have not found any significant differences between elderly colorectal cancer patients and controls in the comorbidity burden (Driver *et al*, 2010). Comorbidity had a negative impact on overall but not on cancer-specific mortality which is in accordance with the findings of most other studies (Yancik *et al*, 1998; Janssen-Heijnen *et al*, 2005; Asmis *et al*, 2008; Zeber *et al*, 2008). However, Koukoran *et al* found that comorbidity was not associated with overall but with an increased cancer-specific mortality. Our findings suggest that elderly colorectal cancer patients do not receive inferior antineoplastic treatment resulting in early death from colorectal cancer. Rather, they die from their comorbid conditions.

Prostate cancer cases experienced more connective tissue and ulcer disease than controls. This has not been described elsewhere and should be confirmed by other studies. It might be speculated that a diagnosis of connective tissue disease is likely to be associated with the use of non-steroidal anti-inflammatory drugs (NSAIDs) that are associated with an increased risk of ulcer disease (Hallas *et al*, 1995). Sixty-seven percent of prostate cancer cases used NSAIDS at diagnosis, compared with 43% of controls (data not shown). Comorbidity influenced overall, but not cancer-specific mortality in prostate cancer cases. This is in accordance with the findings of other studies (Albertsen *et al*, 2011; Berglund *et al*, 2011; Riihimaki *et al*, 2011) and indicates a non-inferior antineoplastic treatment of patients with comorbidity.

Ovarian cancer cases generally died from their cancer disease; overall and cancer-specific mortality was similar. Severe comorbidity seemed to be associated with higher mortality, although this was based on small numbers. In a cohort study of 1995, women with ovarian cancer, Tetsche *et al* (2008) found an association between severe comorbidity and impaired survival over a 10-year period. As in our study, the study lacked information on disease stage. In contrast, Mass *et al* (2005) found treatment modality to be the only parameter with a statistically significant prognostic effect in a population-based study on 1116 ovarian cancer cases, which included information on disease stage, treatment modality, age, and comorbidity.

The strengths of our study are the population-based approach with a large sample size, and use of valid registers with high coverage, minimising the risk of selection bias. The DCR has been found to cover 95–98% of all cancer diagnoses in Denmark (Storm *et al*, 1997). Eighty-seven percent of the tumours are verified histologically, and only 0.5% are registered solely on the basis of death certificates (Danish Health Board, 2009). All diagnoses in FPAS are transferred to the Danish National Hospital Register (DNHR). The DNHR has been validated and shown to possess a high degree of completeness and validity of administrative data (Andersen *et al*, 1999). Furthermore, incorrect or missing diagnoses in FPAS will most likely be equally distributed among cases and controls, and thus result in a non-differential misclassification.

Only few studies have validated the Danish Register of Causes of Death (Pedersen *et al*, 2006). The causes of death are based on death certificates, often filled by the patients’ general physicians. One limitation is that it is possible that for deceases with a previous diagnosis of cancer and an unclear cause of death, the physician may apply the cancer disease as the cause of death. This will result in artificially higher rates of cancer-specific death. In our study, however, comorbidity was not associated with a higher risk of death from cancer.

Limitations of our study are the lack of data on stage of disease and cancer therapy. This might have confounded the effect of comorbidity on cancer-specific mortality. We might have expected patients with a high comorbidity burden to have undergone less intensive treatment. However, we found no general effect of comorbidity on cancer-specific mortality and we thus believe that our results are valid.

In conclusion, diagnoses of colorectal and lung cancer were associated with increased comorbidity burden compared with the background population. Moderate to severe renal disease was more prevalent in elderly cancer cases than in controls, regardless of cancer site. Comorbidity was associated with increased overall mortality of elderly cancer patients, but an association with cancer-specific mortality was only seen for severe comorbidity in lung cancer.

## Figures and Tables

**Table 1:** Characteristics of cases and controls

	**Cases**	**Controls**
*Median age (IQR)*		
All	78 (74–83)	78 (74–83)
Breast cancer (*n*=1106)	79 (74–85)	—
Lung cancer (*n*=1719)	76 (73–80)	—
Colorectal cancer (*n*=2040)	79 (74–84)	—
Prostate cancer (*n*=1231)	78 (74–83)	—
Ovarian cancer (*n*=229)	77 (74–82)	—
		
**CCIS**	***N* (%)**	***N* (%)**
CCIS 0	5192 (82.1)	21 868 (86.4)
CCIS 1–2	779 (12.3)	2428 (9.6)
CCIS 3+	354 (5.6)	1003 (4.0)
		
*History of*		
Ischemic heart disease	127 (2.01)	373 (1.47)
Congestive heart failure	199 (3.15)	548 (2.17)
Periferal vascular disease	147 (2.32)	352 (1.39)
Cerebrovascular disease	172 (2.72)	541 (2.14)
Dementia	54 (0.85)	294 (1.16)
Chronic pulmonary disease	778 (12.3)	2035 (8.04)
Connective tissue disease	46 (0.73)	136 (0.54)
Ulcer disease	137 (2.17)	312 (1.23)
Mild liver disease	12 (0.19)	42 (0.17)
DM	405 (6.40)	1352 (5.34)
Hemiplegia	7 (0.11)	7 (0.03)
Moderate to severe renal disease	44 (0.70)	76 (0.30)
DM with end organ damage	162 (2.56)	513 (2.03)
Moderate to severe liver disease	1 (0.02)	10 (0.04)

Abbreviations: CCIS=Charlson comorbidity index score; DM=diabetes mellitus; IQR=interquartile range.

**Table 2:** Comorbidity and OR for the association between CCI score and cancer status according to cancer site

	**CCIS 0**			**CCIS 1–2**			**CCIS 3+**		
**Cancer site**	**Cases *n* (%)**	**Controls *n* (%)**	**OR**	**Cases *n* (%)**	**Controls *n* (%)**	**OR (95% CI)**	**Cases *n* (%)**	**Controls *n* (*n*%)**	**OR (95% CI)**
Breast	964 (87.2)	3886 (87.9)	Ref.	99 (9.0)	380 (8.6)	1.06 (0.84–1.34)	43 (3.9)	157 (3.5)	1.11 (0.78–1.57)
Lung	1299 (75.6)	5959 (92.2)	Ref.	303 (17.6)	667 (9.7)	2.07 (1.78–2.40)	117 (6.8)	250 (3.6)	2.12 (1.67–2.68)
Colon/rectum	1690 (82.8)	7020 (86.0)	Ref.	234 (11.5)	808 (9.9)	1.19 (1.02–1.39)	116 (5.7)	332 (4.1)	1.50 (1.20–1.87)
Prostate	1037 (84.2)	4199 (85.3)	Ref.	123 (10.0)	490 (10.0)	1.03 (0.83–1.27)	71 (5.8)	235 (4.8)	1.20 (0.91–1.59)
Ovary	202 (88.2)	804 (87.8)	Ref.	20 (8.7)	83 (9.1)	0.98 (0.58–1.64)	7 (3.1)	29 (3.2)	0.93 (0.40–2.15)

Abbreviations: CCI=Charlson's comorbidity index; CCIS=Charlson comorbidity index score; CI=confidence interval; OR=odds ratio; Ref.=reference.

**Table 3:** Overall mortality at 3 months, 1 and 5 years and HR according to cancer site and comorbidity level

**Cancer site, CCIS**	***n***	**3 Months, % (95% CI)**	**1 Year, % (95% CI)**	**5 Years, % (95% CI)**	**HR (95% CI)**
*Breast*	1100				
CCIS=0	959	9 (8–11)	17 (15–20)	48 (45–52)	1.00 (Ref.)
CCIS=1–2	98	11 (6–19)	18 (12–28)	61 (61–71)	1.40 (1.10–1.79)
CCIS⩾3	43	14 (7–28)	21 (11–36)	48 (34–65)	0.93 (0.61–1.43)
					
*Lung*	1702				
CCIS=0	1287	38 (36–41)	73 (71–75)	94 (93–95)	1.00 (Ref.)
CCIS=1–2	299	39 (34–45)	76 (71–81)	95 (92–97)	1.12 (0.99–1.28)
CCIS⩾3	116	49 (40–59)	85 (78–91)	NA	1.51 (1.24–1.83)
					
*Colorectal*	2029				
CCIS=0	1681	24 (22–26)	41 (39–43)	68 (66–70)	1.00 (Ref.)
CCIS=1–2	232	25 (20–31)	44 (38–51)	73 (66–79)	1.18 (1.00–1.38)
CCIS⩾3	116	32 (24–41)	51 (42–60)	78 (70–86)	1.41 (1.14–1.73)
					
*Prostate*	1228				
CCIS=0	1034	9 (7–10)	22 (20–25)	65 (62–68)	1.00 (Ref.)
CCIS=1–2	123	17 (11–25)	31 (24–40)	69 (60–78)	1.19 (0.96–1.47)
CCIS⩾3	71	27 (18–39)	46 (36–59)	84 (74–92)	2.14 (1.65–2.77)
					
*Ovary*	228				
CCIS=0	201	27 (22–34)	53 (47–60)	84 (78–89)	1.00 (Ref.)
CCIS=1–2	20	20 (8–45)	35 (18–60)	87 (67–97)	0.91 (0.56–1.50)
CCIS⩾3	7	43 (16–83)	81 (44–99)	NA	1.39 (0.57–3.42)

Abbreviations: CCIS=Charlson comorbidity index score; CI=confidence interval; HR=hazard ratio; NA=not applicable, OR=odds ratio; Ref.=reference.

**Table 4:** Cancer-specific mortality (95% CI) at 3 months, 1 and 5 years and HR according to cancer site and comorbidity level

**Cancer site, CCIS**	***n***	**3 Months, % (95% CI)**	**1 Year, 5 (95% CI)**	**5 Years, 5 (95% CI)**	**HR (95% CI)**
*Breast*	1095				
CCIS=0	954	6 (4–7)	11 (10–14)	31 (28–35)	1.00 (Ref.)
CCIS=1–2	98	8 (4–16)	13 (7–21)	32 (23–44)	1.18 (0.83–1.68)
CCIS⩾3	43	5 (1–18)	10 (4–25)	16 (8–33)	0.48 (0.21–1.07)
					
*Lung*	1698				
CCIS=0	1284	36 (33–39)	70 (68–73)	92 (90–93)	1.00 (Ref.)
CCIS=1–2	298	36 (31–42)	73 (68–78)	94 (90–96)	1.12 (0.98–1.29)
CCIS⩾3	116	39 (31–50)	80 (71–88)	NA	1.29 (1.03–1.60)
					
*Colorectal*	2019				
CCIS=0	1675	21 (19–23)	36 (34–38)	58 (56–61)	1.00 (Ref.)
CCIS=1–2	229	23 (18–29)	39 (33–46)	59 (52–66)	1.12 (0.93–1.35)
CCIS⩾3	115	20 (14–29)	36 (27–46)	58 (47–69)	1.00 (0.76–1.33)
					
*Prostate*	1219				
CCIS=0	1028	6 (4–7)	16 (14–19)	52 (49–56)	1.00 (Ref.)
CCIS=1–2	123	10 (6–17)	19 (13–28)	49 (39–61)	0.89 (0.67–1.20)
CCIS⩾3	68	16 (9–28)	27 (18–41)	51 (36–69)	1.32 (0.89–1.94)
					
*Ovary*	227				
CCIS=0	200	24 (19–31)	51 (45–59)	82 (76–87)	1.00 (Ref.)
CCIS=1–2	20	20 (8–45)	35 (18–60)	86 (63–97)	0.88 (0.52–1.48)
CCIS⩾3	7	43 (16–83)	81 (44–99)	NA	1.53 (0.62–3.77)

Abbreviations: CCIS=Charlson comorbidity index score; CI=confidence interval; HR=hazard ratio; NA=not applicable, OR=odds ratio; Ref.=reference.
